# Lead Exposure and Cardiovascular Disease—A Systematic Review

**DOI:** 10.1289/ehp.9785

**Published:** 2006-12-22

**Authors:** Ana Navas-Acien, Eliseo Guallar, Ellen K. Silbergeld, Stephen J. Rothenberg

**Affiliations:** 1 Department of Environmental Health Sciences and; 2 Departments of Epidemiology and Medicine, Johns Hopkins Bloomberg School of Public Health, Baltimore, Maryland, USA; 3 Welch Center for Prevention, Epidemiology and Clinical Research, Johns Hopkins University, Baltimore, Maryland, USA; 4 Centro de Investigación y de Estudios Avanzados – Instituto Politécnico Nacional (CINVESTAV-IPN), Mérida, Yucatán, México; 5 Instituto Nacional de Salud Pública, Cuernavaca, Morelos, México

**Keywords:** atherosclerosis, blood pressure, cardiovascular disease, heart rate variability, hypertension, lead, systematic review

## Abstract

**Objective:**

This systematic review evaluates the evidence on the association between lead exposure and cardiovascular end points in human populations.

**Methods:**

We reviewed all observational studies from database searches and citations regarding lead and cardiovascular end points.

**Results:**

A positive association of lead exposure with blood pressure has been identified in numerous studies in different settings, including prospective studies and in relatively homogeneous socioeconomic status groups. Several studies have identified a dose–response relationship. Although the magnitude of this association is modest, it may be underestimated by measurement error. The hypertensive effects of lead have been confirmed in experimental models. Beyond hypertension, studies in general populations have identified a positive association of lead exposure with clinical cardiovascular outcomes (cardiovascular, coronary heart disease, and stroke mortality; and peripheral arterial disease), but the number of studies is small. In some studies these associations were observed at blood lead levels < 5 μg/dL.

**Conclusions:**

We conclude that the evidence is sufficient to infer a causal relationship of lead exposure with hypertension. We conclude that the evidence is suggestive but not sufficient to infer a causal relationship of lead exposure with clinical cardiovascular outcomes. There is also suggestive but insufficient evidence to infer a causal relationship of lead exposure with heart rate variability.

**Public Health Implications:**

These findings have immediate public health implications. Current occupational safety standards for blood lead must be lowered and a criterion for screening elevated lead exposure needs to be established in adults. Risk assessment and economic analyses of lead exposure impact must include the cardiovascular effects of lead. Finally, regulatory and public health interventions must be developed and implemented to further prevent and reduce lead exposure.

## Background

Cardiovascular disease is the leading cause of mortality and a primary contributor to the burden of disease worldwide (Lopez et al. 2006). Environmental toxicants, including lead and other metals, are potentially preventable exposures that may explain population variation in cardiovascular disease rates (Bhatnagar 2006; Weinhold 2004). However, after more than 100 years since initial reports suggested a link between lead exposure and cardiovascular outcomes (Lancéreaux 1881; Lorimer 1886), the contribution of lead to cardiovascular disease is still incompletely understood.

Population research on the cardiovascular effects of lead has focused largely on the association with blood pressure and hypertension. Several reviews and metaanalyses combining data from more than 30 original studies and around 60,000 participants have examined the evidence relating blood lead to blood pressure or hypertension [Hertz-Picciotto and Croft 1993; Nawrot et al. 2002; Schwartz 1995; Sharp et al. 1987; Staessen et al. 1994, 1995; U.S. Environmental Protection Agency (U.S. EPA) 2006]. All these reviews concluded that there was a positive association between blood lead levels and blood pressure (Table 1). The estimated increase in systolic blood pressure associated with a 2-fold increase in blood lead levels (e.g., from 5 to 10 μg/dL) ranged across reviews from 0.6 to 1.25 mmHg. This epidemiologic relationship is also supported by a large body of experimental and mechanistic evidence (U.S. EPA 2006). Because lead exposure is widespread, even a modest effect would imply that lead exposure is an important determinant of blood pressure levels and hypertension in human populations.

The cardiovascular effects of lead, however, are not limited to increased blood pressure and hypertension. Lead exposure has also been associated with an increased incidence of clinical cardiovascular end points such as coronary heart disease, stroke, and peripheral arterial disease (Lustberg and Silbergeld 2002; Menke et al. 2006; Navas-Acien et al. 2004; Schober et al. 2006), and with other cardiovascular function abnormalities such as left ventricular hypertrophy and alterations in cardiac rhythm (Cheng et al. 1998; Schwartz 1991).

In the present article, our objective was to perform a systematic review of the epidemiologic evidence on the association of lead exposure with cardiovascular disease end points. Because previous reviews have examined the connection between lead and blood pressure in depth (Table 1), our systematic review emphasizes other clinical and intermediate cardiovascular outcomes to obtain a broader picture of the impact of lead on cardiovascular disease. Finally, we assessed the causal role of lead on blood pressure and cardiovascular disease by applying the criteria and terminology of the 2004 Surgeon General Report *The Health Consequences of Smoking* [U.S. Department of Health and Human Services (U.S. DHHS) 2004] to the available information.

## Methods

### Search strategy and data abstraction

We aimed to identify all observational studies assessing the association between lead exposure and cardiovascular end points. Using free text and key words (Appendix A), we searched PubMed (http://www.ncbi.nlm.nih.gov/entrez/query.fcgi?db=PubMed), EMBASE (http://www.embase.com/), and TOXLINE (http://toxnet.nlm.nih.gov/) through August 2006 with no language restrictions. In addition we manually reviewed the reference lists from relevant original research and review articles and documents.

For lead exposure, we included studies that used biomarkers (lead levels in blood, bone, or other specimens), environmental measures (airborne lead levels), or indirect measures (job titles, job exposure matrices, living in lead-contaminated areas). For cardiovascular end points, we included studies that reported clinical cardiovascular end points (cardiovascular disease, coronary heart disease, stroke, or peripheral arterial disease) and intermediate cardiovascular end points (left ventricular mass, heart rate, heart rate variability, or electrocardiographic abnormalities) other than blood pressure levels or hypertension.

**Figure 1:**
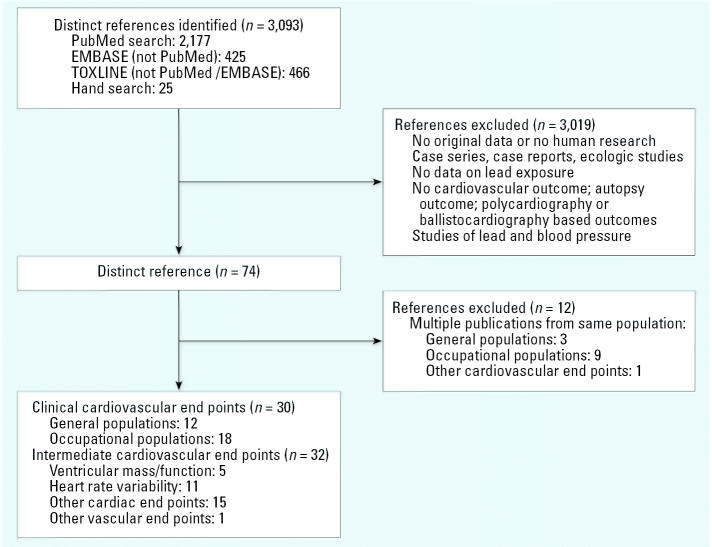
Flow diagram of study selection process. Databases: PubMed (http://www.ncbi.nlm.nih.gov/entrez/query.fcgi?db=PubMed); EMBASE (http://www.embase.com/); TOXLINE (http://toxnet.nlm.nih.gov/).

We excluded publications containing no original research, studies not carried out in humans, case reports, case series, ecologic studies, studies lacking a cardiovascular outcome, and studies lacking data on lead exposure (Figure 1). For studies with multiple publications on the same population, we selected the publication with the longest follow-up. For studies with equivalent follow-up periods, we selected the study with the largest number of cases or the most recent publication. We excluded autopsy studies measuring lead in arterial tissue and studies based on polycardiography and ballistocardiograpy, techniques no longer in use. For consistency, blood lead levels were converted to micrograms per deciliter.

We adapted the criteria used by Longnecker et al. (1988) to assess study quality for studies of clinical end points and the criteria used by Appel et al. (2002) to assess study quality for studies of intermediate end points (Appendices B and C).

### Statistical methods

Measures of association (odds ratios, prevalence ratios, standardized mortality ratios, relative risks, relative hazards, comparisons of means, linear regression coefficients, correlation coefficients) and their standard errors were abstracted or derived from published data (Greenland 1987). For studies reporting measures of association for population subgroups (Cooper et al. 1985; Malcolm 1971), we pooled the measures of association using an inverse-variance weighted random-effects model (Egger et al. 2001).

Because of substantial heterogeneity and methodologic limitations of the original studies, we considered that quantitative pooling was inappropriate. We thus present a qualitative systematic review of the available evidence.

## Results

### Lead and clinical cardiovascular disease in general populations

Twelve studies met our inclusion criteria (Table 2). Lead was measured in blood in all the prospective cohort studies (Kromhout 1988; Lustberg and Silbergeld 2002; Menke et al. 2006; Møller and Kristensen 1992; Pocock et al. 1988) and in the only cross-sectional study available (Muntner et al. 2005). Blood lead levels were substantially lower in more recent compared with older studies. Case–control studies assessed lead exposure on the basis of lead levels in blood (Kosmala et al. 2004), plasma (Mansoor et al. 2000), and urine (Pan et al. 1993; Tsai et al. 2004), on a job exposure matrix (Gustavsson et al. 2001), and on lead levels in the air of the residential neighborhood of study participants (Dulskiene 2003). None of these studies determined lead in bone. Although cohort studies and the cross-sectional study tended to fulfill prespecified quality criteria, case–control studies failed to fulfill some important quality criteria (Appendix B).

Lead exposure was positively associated with clinical cardiovascular end points in all studies (Table 2). Among prospective studies, the relative risks for coronary heart disease ranged between 1.1 comparing blood lead levels > 24.8 μg/dL versus < 12.4 μg/dL in the British Regional Heart Study (Pocock et al. 1988) and 1.89 comparing blood lead levels ≥ 3.63 μg/dL versus < 1.93 μg/dL in the National Health and Nutrition Examination Survey (NHANES) III Mortality Follow-up Study (Menke et al. 2006). The relative risk for stroke in the NHANES III Mortality Follow-up Study was 2.51. There were no prospective studies on the association of blood lead with peripheral arterial disease. However, the relative risk for peripheral arterial disease comparing blood lead levels ≥ 2.47 μg/dL versus < 1.03 μg/dL in a cross-sectional analysis of NHANES 1999–2002 was 1.92 (Muntner et al. 2005).

### Lead and cardiovascular mortality in occupational populations

Eighteen studies from the United States (Cooper et al. 1985; Michaels et al. 1991; Robinson 1974; Sheffet et al. 1982; Steenland et al. 1992; Tollestrup et al. 1995), Europe (Alexieva et al. 1981; Belli et al. 1989; Carta et al. 2003; Cocco et al. 1997, 1994; Davies 1984; Dingwall-Fordyce and Lane 1963; Gerhardsson et al. 1995; Lundstrom et al. 1997; Malcolm 1971; Wilczynska et al. 1998), and Australia (McMichael and Johnson 1982) met our inclusion criteria (Table 3). Battery, ceramic, pigment, refinery, and smelter industries were studied. All studies used job titles to ascertain exposure and death certificates to identify coronary heart disease (12 studies), stroke (15 studies) and overall cardiovascular mortality (9 studies). Most were retrospective cohort studies and used external comparisons to the general population to derive standardized mortality ratios. The exceptions were the study by Dingwall-Fordyce and Lane (1963), two proportional mortality studies (Alexieva et al. 1981; McMichael and Johnson 1982) and two prospective cohort studies (Robinson 1974; Tollestrup et al. 1995). Occupational studies failed to fulfill most prespecified quality criteria (Appendix B).

Relative risk estimates across occupational studies varied widely, with positive, inverse, and null associations (Table 3). Several studies reported the associations among workers with the heaviest exposure (Dingwall-Fordyce and Lane 1963; Lundstrom et al. 1997; Malcolm and Barnett 1982; Steenland et al. 1992), by year of hire (Cooper et al. 1985; Lundstrom et al. 1997), and incorporating a latency period (Lundstrom et al. 1997). In two of the three studies that reported associations by duration of employment, coronary heart disease (Steenland et al. 1992) and stroke (Michaels et al. 1991) mortality were higher among workers with the highest number of years of employment.

### Lead and intermediate cardiovascular outcomes

Five studies evaluated ventricular wall dimensional and functional parameters (Beck and Steinmetz-Beck 2005; Kasperczyk et al. 2005; Schwartz 1991; Tepper et al. 2001; Zou et al. 1995) (Table 4). Increased blood lead levels were associated with an increased prevalence of left ventricular hypertrophy in U.S. adults (Schwartz 1991) and with a nonstatistically significant increase in left ventricular mass in U.S. battery workers (Tepper et al. 2001). Similarly, Polish steel workers had higher left ventricular mass and lower ejection fraction compared to administrative workers from the same factory (Kasperczyk et al. 2005), and lead-exposed Polish workers had impaired diastolic function compared with nonexposed controls (Beck and Steinmetz-Beck 2005). Chinese refinery workers with blood lead levels > 50 μg/dL had similar interventricular septum and left ventricular wall thickness compared to workers < 50 μg/dL (Zou et al. 1995), although lead levels in the reference category are unknown.

Ten studies measured heart rate variability among lead-exposed workers (Andrzejak et al. 2004; Böckelmann et al. 2002; Gajek et al. 2004; Gennart et al. 1992; Ishida et al. 1996; Murata et al. 1995; Murata and Araki 1991; Muzi et al. 2005; Niu et al. 1998; Teruya et al. 1991), and one study measured heart rate variability in Seoul, Korea, public officials not occupationally exposed to lead (Jhun et al. 2005) (Table 4). Most of these studies had limitations in terms of sample size, methods of lead assessment, and lack of adjustment for potential confounders (Table 4; Appendix C). The conditions for electrocardiographic ascertainment and the heart rate variability indices differed widely across studies, making comparisons difficult. The coefficient of variation of the R-R interval was lower in lead-exposed workers compared with other workers in two of five studies in which the coefficient of variation was measured under normal breathing, and in one of three studies in which it was assessed during deep breathing. Among Seoul public officials (Jhun et al. 2005), increased lead levels were inversely associated with measures of low frequency, high frequency, and total power spectrum in uni-variate analyses, but adjusted results were not presented because lead exposure was dropped from the stepwise regression models used.

Fifteen studies reported the association of lead with other electrocardiographic parameters (Cheng et al. 1998; Gatagonova 1995a, 1995c; Kirkby and Gyntelberg 1985; Kosmider 1968; Kosmider and Petelenz 1961, 1962; Kosmider et al. 1965; Kromhout et al. 1985; Krotkiewski et al. 1964; Saric 1981; Shcherbak 1988; Sroczynski et al. 1990, 1985; Stozinic and Colakovic 1980) and one study with other vascular abnormalities (Aiba et al. 1999). All studies, except the Normative Aging Study (Cheng et al. 1998), were conducted in occupational populations in Europe. These types of outcome, including rhythm disorders, ischemic changes and cycle duration, varied widely across studies, and the findings were inconsistent. The Normative Aging Study measured lead in blood, tibia, and patella and identified associations between tibia lead and intraventricular conduction defects (QRS duration) and increased QT duration in subjects < 65 years of age (Cheng et al. 1998).

Finally, heart rate was evaluated using different methods in five studies, four in lead-exposed workers (Böckelmann et al. 2002; Kosmider and Petelenz 1961; Murata et al. 1995; Zou et al. 1995) and one in elderly men from the Netherlands (Kromhout et al. 1985), with inconsistent findings.

## Discussion

### Lead exposure and hypertension—sufficient evidence to infer a causal relationship

Chronic lead poisoning was connected to hypertension in the 19th century (Lorimer 1886). With rare exceptions (Vigdortchik 1935), a major limitation of early reports was the lack of a comparison group (Sharp et al. 1987). The hypertensive effects of lead have been extensively documented in experimental animals chronically exposed to high lead concentrations and in workers chronically exposed to high lead levels (Agency for Toxic Substances and Disease Registry 1999; U.S. EPA 2006). Generally, the development of hypertension in subjects chronically exposed to high lead levels has been interpreted as a possible consequence of lead nephropathy. At environmental levels of exposure, however, the effect of lead on blood pressure has been controversial. Numerous studies have addressed this question. All reviews have concluded that there is an association between lead and blood pressure, although the strength of this association is modest (Table 1). Substantial evidence, however, implies that this relationship is causal.

#### Consistency

The association between lead exposure and blood pressure has been found in populations with different geographic, ethnic, and socioeconomic background. While residual confounding by socioeconomic status is a concern, studies in homogenous samples and studies that have adjusted for a variety of socioeconomic indicators have still identified an association between lead exposure and blood pressure (Martin et al. 2006; Pocock et al. 1984).

#### Temporality

The association between blood lead and elevated blood pressure has been identified not only in cross-sectional but also in prospective studies that showed that new cases of hypertension and within-person elevations in blood pressure levels over follow-up were related to baseline lead exposure (Glenn et al. 2003; Møller and Kristensen 1992; Weiss et al. 1986).

#### Strength of the association

While the strength of the association between lead and blood pressure is modest, it may have been substantially underestimated because of measurement error in both lead and blood pressure determinations. Most studies used single blood lead measurements to assess lead exposure. When bone lead was used as a bio-marker of long-term exposure (Hu et al. 2007), lead in cortical or trabecular bone was positively associated with increased systolic blood pressure or hypertension in all prospective (Cheng et al. 2001; Glenn et al. 2003) and cross-sectional studies (Gerr et al. 2002; Hu et al. 1996; Korrick et al. 1999; Lee et al. 2001; Martin et al. 2006; Rothenberg et al. 2002; Schwartz and Stewart 2000). Furthermore, even bone lead is subject to error derived from the sampling site and from the technical difficulties of the measurement. In addition, blood pressure measurements were often conducted using nonstandardized protocols, without repeated measures, or in samples including hypertensive subjects.

#### Biologic gradient (dose response)

Some studies have demonstrated a progressive dose–response relationship between lead exposure and blood pressure (Pocock et al. 1984; Schwartz 1988; Weiss et al. 1986). However, the shape of the dose–response relationship is not completely characterized, particularly at low levels of exposure. It is not known what is the lowest level of lead exposure not associated with blood pressure, although in the available studies there seems to be no evidence of a threshold effect (Hertz-Picciotto and Croft 1993; Schwartz et al. 2001).

#### Biologic plausibility and experimental data

Numerous experimental studies in animals have shown irrefutable evidence that chronic exposure to low lead levels results in arterial hypertension that persists long after the cessation of lead exposure (U.S. EPA 2006). The precise mechanisms explaining a hypertensive effect of low chronic exposure to environmental lead are unknown. An inverse association between estimated glomerular filtration rate and blood lead has been observed at blood lead levels < 5 μg/dL in general population studies (Ekong et al. 2006; Muntner et al. 2005), indicating that lead-induced reductions in renal function could play a major role in hypertension. Other potential mechanisms include enhanced oxidative stress (Stohs and Bagchi 1995; Vaziri et al. 2001), stimulation of the renin-angiotensin system (Carmignani et al. 1999; Rodriguez-Iturbe et al. 2005), and down-regulation of nitric oxide (Ding et al. 1998; Dursun et al. 2005) and soluble guanylate cyclase (Farmand et al. 2005). These mechanisms could result in increased vascular tone and peripheral vascular resistance (U.S. EPA 2006).

#### Causal inference

We conclude that the evidence is sufficient to infer a causal relationship between lead exposure and high blood pressure. Further research is still needed to determine the precise dose–response relationship, the relative importance of short-term versus chronic lead effects, the relevant mechanisms at environmental levels of exposure, and whether the magnitude of the association is different in children or in other vulnerable population subgroups.

### Clinical cardiovascular end points in general populations

#### Consistency and temporality

Few cohort studies have evaluated the prospective association of lead with clinical cardiovascular outcomes in general population settings. The findings of the NHANES II and NHANES III Mortality Follow-up studies are remarkable. NHANES are periodic, standardized surveys designed to provide representative health data from the U.S. noninstitutionalized population. Despite a marked decline in lead levels in U.S. adults, both surveys showed statistically significant increases in cardiovascular mortality with increasing blood lead (Lustberg and Silbergeld 2002; Schober et al. 2006). In addition a cross-sectional analysis of NHANES 1999–2002 data identified an association of blood lead with the prevalence of peripheral arterial disease (Muntner et al. 2005; Navas-Acien et al. 2004). The British Regional Heart Study (Pocock et al. 1988) and two other small cohort studies (Kromhout 1988; Møller and Kristensen 1992) showed positive but nonstatistically significant associations of coronary heart disease or stroke incidence with higher lead levels. The confidence intervals from these studies were wide but included the point estimates of the NHANES studies. Additional studies are needed to determine the consistency of the evidence in diverse populations.

#### Strength of the association and dose response

The associations of blood lead with clinical cardiovascular end points in the NHANES studies were moderately strong, with a clear dose–response gradient. An unresolved issue is the impact of uncontrolled confounding and measurement error on the relative risk estimates in studies of lead and clinical cardiovascular end points. NHANES studies adjusted for race, education, income, and urban versus rural location, which reduces potential confounding by socioeconomic status. Studies with more detailed information on the determinants of lead exposure may contribute to a better understanding of this issue. Similarly, evaluating lead effects using a single blood lead measure may result in measurement error with substantial underestimation of the magnitude of the association. This is particularly problematic when there are marked temporal trends in lead levels, as this source of error adds to within-person variability in blood lead levels to increase regression-dilution bias.

#### Biologic plausibility and experimental data

Lead levels of 0.8 ppm (Revis et al. 1981) and 0.1 ppm (Minaii et al. 2002) in drinking water induced atherosclerosis in animal models, and lead levels of 0.5–10 μM induced the proliferation of vascular smooth cells and fibroblasts in *in vitro* models (Fujiwara et al. 1995). Lead-related atherosclerosis could be explained by several mechanisms, including increases in blood pressure, impairment of renal function (Ekong et al. 2006), and induction of oxidative stress (Stohs and Bagchi 1995; Vaziri et al. 2001), inflammation (Heo et al. 1996), and endothelial dysfunction (Vaziri et al. 2001).

#### Causal inference

Because of the scarce number of prospective studies and the lack of information on incident nonfatal events, we conclude that the evidence is suggestive but not sufficient to infer a causal relationship with clinical cardiovascular end points. Prospective studies are required to characterize fully the impact of lead on cardiovascular morbidity and mortality. These studies need detailed and repeated assessment of lead exposure and its determinants, standardized assessment of traditional cardiovascular risk factors, and long-term follow-up to identify incident cardiovascular events and trends in subclinical markers of atherosclerosis. Although elevated blood pressure and impaired renal function are proposed mechanisms that mediate the effects of lead on clinical cardiovascular outcomes, other mechanisms are likely to be involved. Future epidemiologic studies should explore in detail the magnitude of the contribution of specific mediators of clinical cardiovascular lead effects.

### Cardiovascular mortality in occupational populations

#### Adequacy of the evidence

The validity of occupational studies of lead and cardiovascular mortality is limited by several methodologic problems. A major limitation is the healthy worker effect (Arrighi and Hertz-Picciotto 1994). The comparison of exposed workers with the general population is particularly inappropriate for cardiovascular mortality because workers are healthier and their lifestyles and cardiovascular risk factors are likely to differ widely from those of the general population (Choi 1992). In addition, cardiovascular diseases are associated with prolonged disability and changes in employment status. Even in studies based on comparisons with unexposed workers, the selection of healthier individuals at time of hire or for specific jobs within an industry may have resulted in biased estimates of the association. Correcting the bias introduced by the healthy worker survivor effect is extremely challenging, and stratifying by duration of employment or time since hire is unlikely to completely account for this source of bias (Arrighi and Hertz-Picciotto 1994; Howe et al. 1988).

Additional limitations include the assignment of lead exposure based on job titles and of cardiovascular deaths based on death certificates. Misclassification of exposure and outcome may have resulted in further underestimation of the association of lead and cardiovascular end points. Finally, the lack of determinations of established cardiovascular risk factors and of other occupational exposures may have contributed to uncontrolled confounding.

#### Causal inference

As a result of these methodologic limitations, and despite many occupational cohort studies published in the literature (Table 3), available information on occupational lead exposure and cardiovascular mortality is inadequate to infer the presence or absence of a causal relationship. Because studies of environmental lead exposure provide evidence of an association between lead and cardiovascular mortality at lower exposures than those experienced by occupationally exposed workers, we expect the impact of lead in exposed workers to be at least as important as in environmentally exposed subjects.

### Lead exposure and heart rate variability

#### Consistency, temporality, and strength of the association

Several studies, mostly cross-sectional, found an association between increased lead exposure and decreased heart rate variability. The diversity in the methods and conditions used for measuring heart rate variability makes it difficult to compare the association of lead exposure and heart rate variability across studies. In addition, the validity and precision of these studies are often limited by small sample sizes, limitations in the assessment of lead exposure, and lack of control for established cardiovascular risk factors and other confounders.

#### Biologic plausibility and experimental data

Lead, a well-established neurotoxicant, could affect heart rate variability by interfering in autonomic nervous control of the heart (Chang et al. 2005). Heart rate variability measures the fluctuation of the heart rate around the mean heart rate (Task Force of the European Society of Cardiology and the North American Society of Pacing and Electrophysiology 1996). Because the basis of normal cardiac autonomic functioning is the shift from parasympathetic to sympathetic modulation, decreased heart rate variability is a marker of cardiac autonomic dysfunction. Indeed, decreased heart rate variability in supine position and in response to postural change has been associated with increased incident coronary heart disease and all-cause mortality in large prospective cohort studies in populations free of cardiovascular disease (Liao et al. 1997; Tsuji et al. 1996).

#### Causal inference

We conclude that the evidence is suggestive of but not sufficient to infer a causal relationship of lead exposure with heart rate variability. Large studies with adequate measures of lead exposure and of heart rate variability are needed to better characterize the association between lead exposure and autonomic cardiac control.

### Public health implications

The evidence in this systematic review is sufficient to infer a causal relationship of lead exposure with elevated blood pressure, and it is suggestive of but not sufficient to infer a causal relationship of lead with clinical cardiovascular outcomes and cardiovascular function tests. These associations have been observed at blood lead levels well below 5 μg/dL (Menke et al. 2006; Nawrot and Staessen 2006). Indeed, no lower threshold has been established for any lead-cardiovascular association.

Although future research will contribute to characterize fully the impact of lead exposure on cardiovascular health, these findings have several important public health implications. First, there is an immediate need to lower the current safety standard of the World Health Organization and the U.S. Occupational Safety and Health Administration for blood lead in workers (currently established at 40 μg/dL). Second, a criterion for elevated blood lead levels in adults needs to be established and screened for in preventive services. In fact, the cardiovascular end points described above plus the substantial evidence that chronic lead exposure affects cognitive function (Shih et al. 2007) and renal function (Ekong et al. 2006) at levels < 5 μg/dL indicate that the U.S. Centers for Disease Control and Prevention criterion for elevated blood levels in children (10 μg/dL) is too high for adults. Third, the hypertensive effects of lead exposure and its impact on cardiovascular mortality need to be included in risk assessment and in economic analyses of lead exposure impact. Finally, regulatory and public health interventions must be developed and implemented to prevent and reduce lead exposure beyond current levels in adults.

## Figures and Tables

**Table 1:** Reviews of the association between blood lead levels and blood pressure.

First author, year	Type^a^	No. of studies included	Year of publication of studies (range)	Language of literature search	Total no. of subjects	Age range of participants (years)	Comparison	Outcome	Pooled estimate [change in mmHg (95% CI)]	Median of estimates [change in mmHg (range)]	Conclusions as reported by authors
Sharp et al. 1987	Review	4	1982–1986	English, French	8,406	24–59	Per 2-fold ↑^b^	SBP	—	1.9 (0.7 to 2.3)	Evidence consistent with causation
Hertz-Picciotto and Croft 1993	Review	13	1980–1992	English	22,923	12–80	≠ for each study	SBP DBP Hypertension	— — —	2.0 (−5.9 to 8.0) 1.7 (−1.6 to 4.0) RR: 1.4 (1.2 to 1.7)	Evidence strongly supports causal association
Staessen et al. 1994, 1995	SR, MA	23	1980–1993	English, French, German	33,141	10–88	Per 2-fold ↑	SBP DBP	1.0 (0.4–1.6) 0.6 (0.2–1.0)	1.0 (−3.0 to 14.0) 1.0 (−2.0 to 13.0)	MA suggests a weak association
Schwartz 1995	SR, MA	15	1985–1993	English	NR Men only	18–76	Per 2-fold ↑^b^	SBP	1.25 (0.87–1.63)	1.45 (0.2 to 3.2)	MA consistent with causal association
ATSDR 1999	SR	24	1980–1996	No language restriction	NR	All ages	≠ for each study	SBP DBP Hypertension	— — —	NR NR NR	Suggestion of ↑ blood pressure, but evidence is inconclusive
Nawrot et al. 2002	SR, MA	31	1980–2001	English, French, German	58,518	10–90	Per 2-fold ↑	SBP DBP	1.0 (0.5–1.4) 0.6 (0.4–0.8)	1.0 (−5.0 to 14.0) 1.0 (−2.0 to 14.0)	MA suggests a weak association
U.S. EPA 2006	SR, MA	9 10	1990–2003	English	27,424 34,740	14–93	Per 2-fold ↑	SBP DBP	0.81 (0.46–1.16)^c^ —	1.0 (−3.9 to 11) 1.0 (−1.3 to 7.3)	MA suggests an effect of blood lead on SBP

Abbreviations: ≠, different; ↑, increase; CI, confidence interval; DBP, diastolic blood pressure; MA, meta-analysis; NHANES, National Health and Nutrition Examination Survey; NR, not reported; RR, relative risk; SBP, systolic blood pressure; SR, systematic review; U.S. DHHS, U.S. Department of Health and Human Services; U.S. EPA, U.S. Environmental Protection Agency.

a Systematic review: a search strategy and criteria for manuscript selection are specified. Meta-analysis: a pooled analysis using meta-analysis techniques are presented.

b In the study by Sharp et al. (1987), we divided by 3 the change per 15 μg/dL (equivalent to comparing 10 μg/dL vs. 5 μg/dL). The study by Schwartz et al. (1995) reports the change in mmHg comparing 10 μg/dL vs. 5 μg/dL.

c Pooled estimate using an inverse variance weighted random-effects model (Egger et al. 2001) of two pooled estimates for linear and log-linear estimates, respectively.

**Table 2:** Epidemiologic studies of lead exposure and clinical cardiovascular disease in general populations.

First author, year	Country	Population	Men (%)	Age range (years)	Lead assessment	Range of lead levels	End point ascertainment	Outcome	No. of cases/noncases	Measure of^b^ association	Comparison	Adjusted for^c^
Prospective cohort studies												
Pocock et al. 1988	U.K.	British Regional Heart Study	100	40–49	Blood (AAS)	< 6.2 to > 35.2 μg/dL	Death certificate or chest pain, enzyme, ECG^a^	CHD, F + NF	316/7,063	OR 1.1 (0.4–1.8)	> 24.8 vs. < 12.4 μg/dL	Age, smoking, location
							Death certificate, medical record	Stroke, F + NF	66/7,313	Mean 16.7 μg/dL Mean 15.3 μg/dL	Cases vs. noncases	Age, smoking, location
Kromhout 1988	Netherlands	Elderly men in Zutphen	100	57–76	Blood (AAS)	< 10.8 (10th p) > 28.0 (90th p) μg/dL	Death certificate or chest pain, enzyme, ECG^a^	CHD, F + NF	26/115	HR 1.34 (0.46–3.94)	> 23.8 vs. < 13.0 μg/dL	Age, smoking, BMI, BP, cholesterol
Møller and Kristensen 1992	Denmark	Survey repondents 4 municipalities	48	40	Blood (AAS)	2 to 60 μg/dL	Death certificate, hospital admissions	CHD, F + NF CVD, F + NF	40/1,005 54/991	HR 1.58 (0.85–2.95) HR 1.10 (0.63–1.93)	Per log unit change	Sex, smoking, alcohol, BP, cholesterol, exercise
Lustberg and Silbergeld 2002	U.S.	NHANES II	47	30–74	Blood (AAS)	< 10 to 29 μg/dL	Death certificate	CVD, F	424/3,766	HR 1.39 (1.01–1.91)	20–29 vs. < 10 μg/dL	Age, sex, race, educ., income, smoking, BMI, exercise, location
Menke et al. 2006	U.S.	NHANES III	47	≥ 17	Blood (AAS)	< 1 to 10 μg/dL	Death certificate	CVD, F CHD, F Stroke, F	766/13,198 367/13,597 141/13,823	HR 1.55 (1.08–2.24) HR 1.89 (1.04–3.43) HR 2.51 (1.20–5.26)	< 1.93 vs. ≥ 3.63 μg/dL	Age, sex, race, educ., income, smoking, alcohol, BMI, exercise, cholesterol, CRP, urban residence, menopause, hypertension, kidney function
Case–control and cross-sectional studies												
Pan et al. 1993	Taiwan	Clinic-based	69	NR	Urine (DPASV)	7.9 to 138.4 μg/L	NR	BFD prev.	16/16	30.8 (30.1) μg/L 17.4 (5.4) μg/L	Cases vs. noncases	Age, sex
Mansoor et al. 2000	Sweden	Clinic-based	53	Mean 46	Plasma (TRXFS)	Mean 3.3 ng/g plasma	Angiograms	PAD prev.	65/65	3.3 (0.4) ng/g plasma 3.2 (0.3) ng/g plasma	Cases vs. noncases	Age, sex
Gustavsson et al. 2001	Sweden	SHEEP Study	68	45–70	JEM	NM	Chest pain, ECG enzyme^a^	AMI inc., NF	1,335/1,658	OR 1.03 (0.64–1.65)	≥ 0.04 mg/m^3^ vs. unexp.	Age, sex, smoking, alcohol, BP, BMI, exercise, location
Dulskiene 2003	Lithuania	Clinic-based	100	25–64	Airborne	NM	Medical records	AMI	579/1,777	OR 1.12 (0.76–1.40)	> 0.225 vs. ≤ 0.225 μg/m^3^	Age, sex, smoking, BP
Tsai et al. 2004	Taiwan	Clinic-based	57	NR	Urine (AAS)	5.3 to 123.6 μg/L	NR	BFD prev.	68/68	33.7 (24.3) μg/L 22.2 (11.8) μg/L	Cases vs. noncases	Age, sex
Kosmala et al. 2004	Poland	Clinic-based	53	Mean 62	Blood (AAS)	Mean 3.9 μg/dL	Coronariography, treadmill exercise text	Effort angina	33/18	3.9 (1.4) μg/dL 3.7 (1.2) μg/dL	Cases vs. noncases	Crude
Muntner et al. 2005	U.S.	NHANES 1999–2002	47	≥ 40	Blood (AAS)	< 0.3 to > 10 (98th p) μg/dL	Ankle-brachial BP index	PAD prev.	NR	OR 1.92 (1.02–3.61)	≥ 2.47 vs. < 1.06 μg/dL	Age, sex, race, educ., insurance, smoking, alcohol, BMI, diabetes

Abbreviations: AAS, atomic absorption spectrometry; AMI, acute myocardial infarction; BFD, black foot disease, a form of peripheral arterial disease endemic in the arseniasis areas of southwestern Taiwan; BMI, body mass index; BP, blood pressure levels or hypertension; CHD, coronary heart disease; CI, confidence interval; CVD, cardiovascular disease; DPASV, differential pulse anodic stripping voltammetry; ECG, electrocardiogram; educ., education; F, fatal; F+NF, fatal and nonfatal; HR, hazard ratio; inc., incidence; JEM, job exposure matrix; NF, nonfatal; NHANES, National Health and Nutrition Examination Survey; NM, not measured; NR, not reported; OR, odds ratio; PAD, peripheral arterial disease; p, percentile; prev, prevalence; SHEEP, Stockholm Heart Epidemiology Study; TRXFS, total-reflection X-ray fluorescence spectrometry; unexp., unexposed.

a Standard World Health Organization criteria for myocardial infarction.

b For studies that categorized lead exposure, we report the HR or OR (with 95% CI in parentheses) comparing the highest with the lowest lead category. Otherwise, we present the mean (SD) lead levels for cases and noncases.

c Blood pressure–unadjusted relative risk is as follows: *a*) Menke (2006): cardiovascular mortality 1.64, coronary heart disease mortality 2.01, stroke mortality 2.61; *b*) Gustavsson( 2001): acute myocardial infarction 1.17.

**Table 3:** Epidemiologic studies of cardiovascular mortality in occupational populations exposed to lead.

First author, year	Country	Population	Men (%)	Age range (years)	Outcome	Follow-up (years)	No. of deaths^a^	RR (95% CI)^b^	Comparison	Adjusted for	Corrected for healthy worker effect
Prospective cohort studies											
Robinson 1974	U.S.	Tetraethyl lead production workers	100	20–58	CVD	20	57 *n* = 1,252	0.64 (0.54–0.75)	Production vs. maintenance workers	Crude	No
Tollestrup et al. 1995	U.S.	Orchard workers (lead arsenate)	66	8 to ≥ 55	CHD Stroke	45	NR NR *n* = 1,097	1.27 (0.72–2.23) 0.82 (0.31–2.12)	Workers vs. general population	Age, sex	No
Retrospective cohort studies											
Dingwall-Fordyce and Lane 1963	U.K.	Lead pensioners and workers	100	≥ 65 Mean 55	Stroke	35	51	2.73 (1.31–5.71)^c^	Assembly, plumbers, plate cutting, etc. vs. office, chemist, etc.	Age, period	No
Malcolm 1971, Malcolm and Barnett 1982	U.K.	Lead battery and smelter pensioners and workers	99	< 65 to ≥ 65 at death	CHD Stroke	10 51	99 103	1.00 (0.82–1.22) 1.31 (0.66–1.91)	Workers vs. general population High exposed vs. no exposed	Age	No
Sheffet et al. 1982	U.S.	Pigment plant workers	100	Mean 27.8	CVD^d^	31	139	0.62 (0.52–0.73)	Workers vs. general population		
Davies 1984	U.K.	Pigment plant workers Pigment plant workers + lead poisoning	100 100	18–59 18–59	Stroke Stroke	30 30	31 9	0.94 (0.66–1.33) 4.10 (2.12–7.86)	Workers vs. general population Workers vs. general population	Age, period Age, period	No No
Cooper et al. 1985	U.S.	Lead battery and producing workers	100	< 25–74	CVD CHD Stroke	24	984 715 172	0.97 (0.99–1.06) 0.85 (0.69–1.05) 1.06 (0.76–1.48)	Workers vs. general population	Age (~ findings by year of hire and employment duration)	Partially^e^
Belli et al. 1989	Italy	Lead miners	100	NR	CVD	36	82	0.95 (0.76–1.10)	Workers vs. general population	Age	No
Michaels et al. 1991	U.S.	Newspaper print workers	100	19–83	CHD Stroke	23	186 43	0.63 (0.54–0.73) 1.35 (0.98–1.82)	Workers vs. general population	Age (for stroke, analysis by employment duration^f^)	Partially
Steenland et al. 1992	U.S.	Smelter workers	100	NR	CHD Stroke CHD Stroke	39 39	320 74 239 53	0.94 (0.84–1.05) 1.05 (0.82–1.32) 0.99 (0.87–1.12) 1.05 (0.79–1.37)	Workers vs. general population High exposed vs. general population	Age, period (+ analyses by employment duration^g^) Age, period	Partially
Cocco et al. 1994	Italy	Lead miners	100	Mean 27.7	CVD	28	258	0.63 (0.56–0.72)	Workers vs. general population	Age, period (~ findings for surface and underground workers)	No
Gerhardsson et al. 1995	Sweden	Smelter workers	100	NR	CHD Stroke	20	34 0	1.72 (1.20–2.42) 0 (0.00–1.23)	Workers vs. general population	Age, period (~ findings by year of hire)	No
Lundström et al. 1997	Sweden	Smelter workers	100	15 to ≥ 75 at death	CVD CHD Stroke	32	234 152 36	0.90 (0.80–1.00) 0.80 (0.70–1.00) 0.80 (0.60–1.20)	Workers vs. general population	Age, period (~ findings for highest exposure group and adding a latency period)	No
Cocco et al. 1997	Italy	Smelter workers	100	Mean 30.4	CVD CHD Stroke	48	251 49 105	0.70 (0.62–0.80) 0.34 (0.25–0.45) 0.95 (0.77–1.15)	Workers vs. general population	Age, period	No
Wilczynksa et al. 1998	Poland	Workers compensated for lead poisoning	100	< 29 to ≥ 50 at 1st episode	CVD CHD Stroke	22	231 98 33	0.91 (0.80–1.04) 0.96 (0.78–1.17) 1.03 (0.71–1.45)	Workers vs. general population	Age (~ findings by number of lead poisoning episodes)	No
Carta et al. 2003	Italy	Smelter workers	100	NR	CVD	29	28	0.80 (0.56–1.16)	Workers vs. general population	Age	No
Proportional mortality study											
Alexieva et al. 1981	Bulgaria	Smelter workers	100	Mean at death 61	CHD Stroke	10	26 47	5.60 (1.68–18.6) 0.17 (0.08–0.36)	Workers vs. general population	Age	No
McMichael and Johnson 1982	Australia	Smelter workers	100	30 to > 60 at death	CHD Stroke	40	231 53	0.95 (0.67–1.35) 1.45 (0.76–2.76)	Exposed workers vs. staff workers	Age	No

Abbreviations: CHD, coronary heart disease; CI, confidence interval; CVD, cardiovascular; RR, relative risk; SMR, standard mortality ratio.

In all studies, lead exposure was determined through job titles, and mortality outcomes were assigned through information in death certificates.

a Sample size not available in most studies.

b Relative risk estimates came from SMRs except Robinson (1974) (RR), Tollestrup (1995) (HR), Alexieva (1981) (proportional mortality rate), and McMichael (1982) (proportional mortality rate).

c The within-cohort relative risk was estimated by comparing standardized mortality ratios in the highest versus the lowest category of exposure.

d A total of 15% of subjects with unknown cause of death in death certificate.

e Partial adjustment indicates that authors conducted additional analyses by employment duration.

f For Michaels et al. (1991), SMRs (95%CI) for stroke by number of years of employment are < 10 years, 2.52 (0.06–13,93); 10–19 years, 0.32 (0.01–1.74); 20–29 years, 0.65 (0.18–1.68); ≥ 30 years, 1.68 (1.18–2.31).

g For Steenland et al. (1992), SMRs by numbers of years of employment are as follows: *a*) CHD: 1–5 years, 1.02; 5–20 years, 0.92; ≥ 20 years, 0.86. *b*) Stroke: 1–5 years, 0.83; 5–20 years, 1.01; ≥ 20 years, 1.41.

**Table 4:** Epidemiologic studies of lead exposure and intermediate cardiovascular end points.

First author, year	Country	Population	Sample size (no.)	Men (%)	Age range (years)	Lead assessment	Range levels (μg/dL)	Comparison	End point ascertainment	Main findings
Studies of ventricular mass and function										
Schwartz 1991	U.S.	NHANES II	< 9,932	~ 50	25–74	Blood	NR	Per 1 μg/dL	ECG (Minnesota code)	↑ prevalence left ventricular hypertrophy OR adjusted for age, sex, race = 1.33 (95% CI, 1.09–1.61)
Zou et al. 1995	China	Refinery workers	41	81	24–45	Blood	Mean 42.5	> 50 vs. < 50 μg/dL	US (dimensional and functional parameters)	~ end-diastolic, systolic internal dimension, wall thickness ~ ejection fraction (%), cardiac output (mL/sec), index (mL/sec × m^2^ ~ heart rate
Tepper et al. 2001	U.S.	Battery workers	108	51	36–73	Blood	12–50	34–50 vs. 12–25 μg/dL	US and ECG	↑ left ventricular mass (g/m^2^) but NS (*p* = 0.20)
Kasperczyk et al. 2005	Poland	Steel workers	143	NR	Mean 44	Blood	Mean 23.4	Administrative workers	US (dimensional and functional parameters)	↑ left ventricular mass (g and g/m^2^) ↑ left, ~ right end-diastolic internal dimensions ~ wall thickness (interventricular septum, posterior wall, others) ↓ ejection fraction (%)
Beck and Steinmetz-Beck 2005	Poland	Lead workers	104	100	32–56	Blood	19.3–79.8	Lead exposed vs. control	Echo-doppler	↓ early mitral inflow peak velocity, ↑ late mitral inflow peak velocity ↓ time velocity integral of early vs. late diastolic inflow ~ time velocity integral of early vs. total diastolic inflow ↑ time velocity integral of late vs. total diastolic inflow ~ Isovolumetric relaxation time of left ventricle
Studies of heart rate variability										
Murata and Araki 1991	Japan	Gun workers	32	100	23–58	Job title	< 16–60	Other workers no lead exp.	ECG: 100 R-R intervals, normal breath	↓ CV of R-R interval; ~ CV of LF component, ↓ CV of HF component
Teruya et al. 1991	Japan	Battery, refinery workers	172	100	18–57	Blood	5–76	Correlation, > 50 vs. < 20 μg/dL	ECG: 1 min, normal, deep breath	~, ↓ mean; ~, ↓ SD; and ~, ↓ CV of R-R interval ~, ↓ maximal variation ratio (min/max R-R interval) ~, ↓ maximal variation rate ([min/max R-R interval]/mean)
Gennart et al. 1992	Belgium	Battery workers	183	100	22–55	Blood	4.4–75	Other workers (finishing, maintenance, etc.)	ECG: normal, deep breath	~ CV of R-R interval, ~ CV of mean square of successive differences, and ~ CV of mean ratio of shortest to longest R-R
Murata et al. 1995	Japan	Glass workers	51	0	21–35	Job title	NR	Textile workers	ECG: 100 R-R intervals, normal breath	~ heart rate ↓ CV of R-R interval, ↓ CV of LF and ↓ HF components ↓ LF/HF ratio
Ishida et al. 1996	Japan	Ceramic painters	128	45	29–75	Blood	2.1–69.5	> 30 vs. < 10 μg/dL	ECG: 100 R-R intervals, normal, deep breath Doppler: finger blood flow	~ CV of R-R interval ↓ flow between supine and standing/supine ~ flow drop velocity (supine flow/time to the nadir after standing)
Niu et al. 1998	China	Lead-exposed workers	302	NR	20–59	Job title	NM	Healthy controls	ECG: deep breath, valsalva, stand up	~ R-R interval
Böckelmann et al. 2002	Germany	Lead, iron, steel workers	136	100	Mean 43	Blood	Mean lead workers 31.2	Iron steel workers	ECG: 90 min, 10-step battery test	↓ heart rate at rest ↑ sinus arrhythmia at rest Lack of recovery of LF and HF after test
Gajek et al. 2004	Poland	Foundry workers	35	100	Mean 42	Blood	< 3.6 to > 41.0	Healthy controls	ECG: 24 hr, long- and short-term	~ mean R-R, SDNN, SDNN index, SDANN, rMSSD, pNN50 Short-term only: ~ TP, VLF, LF, HF, LF/HF, HF night / HF day
Andrzejak et al. 2004	Poland	Copper smelter workers	86	100	Mean 43	Blood	Mean lead workers,br>46.8	Healthy controls matched on age, sex, smoking, lipids, BMI	ECG: 24 hr	~ heart rate Long-term: ↓ pNN50, ~ mean R-R, SDNN, SDNN index, SDANN, rMSSD Short-term: all parameters ↓ included LF and HF, except mean R-R and LF:HF
Muzi et al. 2005	Italy	Battery workers	78	96	Mean 38	Blood	< 3.5 to > 31.6	Other workers	ECG: battery tests	↓ R-R interval ratios for lying-standing, lying-standing-lying, deep breaths, and valsalva
Jhun et al. 2005	Korea	Public officials and family	331	55	Mean 38	Blood	< 1.39 to > 3.45	Per natural-log unit	ECG: 3 min, seated position	↓ LF, HF, and total power spectrum
Studies of other cardiac function abnormalities										
Kosmider and Petelenz 1961	Poland	Lead-poisoned workers	140	100	18–45	Job title symptoms	NM	Healthy controls	ECG	↓ heart rate, ↓ P-Q interval ↑ heart muscle lesions and vegetative disorders
Kosmider and Petelenz 1962	Poland	Lead-poisoned workers	76	100	46–65	Job title symptoms	NM	Healthy controls	ECG	↑ heart muscle lesions and vegetative disorders
Krotkiewski et al. 1964	Poland	Lead-poisoned workers	591	78	20–68	Job title symptoms	NM	Other workers	ECG	↑ prevalence of ischemic changes: 32% vs. 13%
Kosmider et al. 1965	Poland	Lead-poisoned workers	100	100	20–45	Job title symptoms	NM	Healthy controls	ECG	↑ heart muscle lesions and vegetative disorders
Kosmider 1968	Poland	Lead-poisoned workers	216	100	18–65	Job title symptoms	NM	Healthy controls	ECG	↑ heart muscle lesions and vegetative disorders
Stozinic and Colakovic 1980	Yugoslavia	Lead-poisoned workers	1,000	100	NR	Job title symptoms	NM	Healthy controls	ECG questionnaire	↑ electrocardiographic abnormalities (including ↑ S-T segment) ↑ self-reported coronary heart disease and intermittent claudication
Saric 1981	Croatia	Residents near to and far from a smelter	502	50	26–70	Area of residency	NM	Residents far from smelter	ECG	~ electrocardiographic abnormalities
Kromhout et al. 1985	Netherlands	Elderly men in Zutphen	152	100	57–76	Blood	< 10.8 > 28.0	Correlation	ECG	~ resting heart rate
Kirkby and Gyntelberg 1985	Denmark	Lead smelter workers	190	89	30–60	Job title	Mean 31	Healthy controls residents in Glostrup	ECG (Minnesota code)	↑ prevalence of ischemic changes: 20% vs. 6%
Sroczynski et al. 1985	Poland	Lead workers	250	100	Mean 41	Job title	NM	Other workers	ECG (Minnesota code)	↑ prevalence of ischemic changes: 10.0% vs. 5.3% ↑ prevalence of rhythm disorders: 14% vs. 2.7%
Shcherbak 1988	Russia	Lead workers	320	100	20–59	Job title	NM	Other workers	ECG	↑ prevalence of ischemic changes: 11.6% vs. 6.7%
Sroczynski et al. 1990	Poland	Lead workers	711	100	20–60	Job title	NM	Other workers	ECG (Minnesota code)	↑ prevalence systolic murmur and rhythm disorders ↑ prevalence ventricular repolarization ~ prevalence of ischemic changes
Gatagonova 1995a,b,d	Russia	Lead workers	500	78	20–60	Job title	Mean 67	Other workers	Integral rehography ECG	Changes of intracardial and peripheral hemodynamics Disorders of myocardial bioelectric activity and contractility ↑ P wave and QT, QRS interval; ~ P–Q interval
Gatagonova 1995c	Russia	Lead workers	68	100	NR	Job title	NM	Other workers	Exercise stress test	↑ prevalence of ischemic changes (↑ S-T segment > 1 mm 15.9 vs. 4.2%)
Cheng et al. 1998	U.S.	NormativeAging Study	775	100	48–93	Blood Tibia Patella	Mean 5.79 Mean 22 μg/g Mean 31 μg/g	Per 10 unit ↑	ECG	Subjects < 65 years: ↑ QT, ↑ QRS interval for tibia and patella, ~ for blood Subjects ≥ 65 years: ~ QT, ~ QRS interval for all biomarkers ~ conduction defects and arrhythmia for all biomarkers, indices and age groups, except ↑ intraventricular conduction defect for tibia lead in < 65 years
Studies of other vascular function abnormalities										
Aiba et al. 1999	Japan	Refinery workers	48	100	18–69	Job title	Mean 43.2	Correlation	Acceleration plethysmography	↓ amplitude ratio of the second/first systolic wave (age adjusted) ~ amplitude ratio of the third/first and third/first waves (age adjusted)

Abbreviations: ↑, ↓ – indicate increase or decrease (statistically significant at *p* < 0.05, unless otherwise specified). BMI, body mass index; CI, confidence interval; CV, coefficient of variation; DB, deep breathe; ECG, electrocardiogram; exp., exposed; HF, high frequency; HRV, heart rate variability; LF, low frequency; NM, not measured; NR, not reported; NS, not significant; OR, odds ratio; pNN50, proportion of interval differences of successive normal-to-normal intervals > 50 msec; RMSSD, square root of the mean-squared differences of successive NN intervals; SD, standard deviation; SDANN, SD of the average normal-to-normal interval. SDNN, SD of the normal-to-normal interval; TP, total power; US ultrasound; V, ventricular; VLF, very low frequency.

## Appendix A. Search strategy

### Free text and key word

Lead, lead poisoning, heavy metals, mortality, atherosclerosis, cardiovascular disease, peripheral arterial disease, peripheral vascular disease, hypertension, blood pressure, heart rate, electrocardiogram, left ventricular hypertrophy.

### Search in PubMed

(Lead [MH] OR Lead poisoning [MH] OR (Metals, heavy [MH] NOT (Actinium OR Americium OR Antimony OR Barium OR Berkelium OR Bismuth OR Californium OR Cesium OR Chromium OR Cobalt OR Copper OR Curium OR Einsteinium OR Fermium OR Francium OR Gallium OR Germanium Gold OR Hafnium OR Indium OR Iridium OR Iron OR Lawrencium OR Manganese OR Molybdenum OR Neptunium OR Nickel OR Niobium OR Nobelium OR Osmium OR Palladium OR Platinum OR Plutonium OR Protactinium OR Radium OR Rhenium OR Rhodium OR Rubidium OR Ruthenium OR Silver OR Strontium OR Tantalum OR Technetium OR Thallium OR Thorium OR Tin OR Tungsten OR Uranium OR Vanadium OR Zinc OR Zirconium))) AND (Cardiovascular Disease [MH] OR Mortality OR Myocardial Infarction OR Stroke OR Peripheral Arterial Disease OR Peripheral Vascular Disease OR Hypertension OR Blood pressure OR Systolic OR Diastolic OR Atherosclerosis OR Arteriosclerosis OR Electrocardiography OR Heart Rate OR Ventricular Hypertrophy OR heart failure)

### Search in EMBASE

(Lead:de OR (Lead poisoning:de)) AND ((cardiovascular disease:de) OR mortality:ti,ab OR (Myocardial Infarction:ti,ab) OR Stroke:ti,ab OR (Peripheral Arterial Disease:ti,ab) OR (Peripheral Vascular Disease:ti,ab) OR Hypertension:ti,ab OR (Blood pressure:ti,ab) OR Systolic:ti,ab OR Diastolic:ti,ab OR Atherosclerosis:ti,ab OR Arteriosclerosis:ti,ab OR Electrocardiography:ti,ab OR (Heart Rate:ti,ab) OR (Ventricular Hypertrophy:ti,ab) OR (heart failure:ti,ab))

### Search in TOXLINE

(Lead [MH] OR Lead poisoning [MH]) AND (Cardiovascular Disease [MH] OR Mortality OR Myocardial Infarction OR Stroke OR Peripheral Arterial Disease OR Peripheral Vascular Disease OR Hypertension OR Blood pressure OR Systolic OR Diastolic OR Atherosclerosis OR Arteriosclerosis OR Electrocardiography OR Heart Rate OR Ventricular Hypertrophy OR heart failure)

## Appendix

**Appendix B:** Criteria for evaluating the design and data analysis of epidemiologic studies of lead exposure and clinical cardiovascular disease.^a^

	General populations												Occupational populations																	
	Cohort studies					CC and CS studies							Prosp.		Retrospective cohort studies														PMS	
	Pocock et al. 1988	Kromhout 1988	Møller and Kristensen 1992	Lustberg and Silbergeld 2002	Menke et al. 2006	Pan et al. 1993	Mansoor et al. 2000	Gustavsson et al. 2001	Dulskiene 2003	Tsai et al. 2004	Kosmala et al. 2004	Muntner et al. 2005	Robinson 1974	Tollestrup et al. 1995	Dingwall-Fordyce and Lane 1963	Malcolm 1971, Malcolm and Barnett 1982	Sheffet et al. 1982	Davies 1984	Cooper et al. 1985	Belli et al. 1989	Michaels 1991	Steenland et al. 1992	Cocco et al. 1994	Gerhardsson et al. 1995	Lundstrom et al. 1997	Cocco et al. 1997	Wilczynksa et al. 1998	Carta et al. 2003	Alexieva et al. 1981	McMichael and Johnson 1982
All studies																														
Lead exposure was assessed at the individual level	Y	Y	Y	Y	Y	Y	Y	Y	N	Y	Y	Y	N	N	N	N	N	N	N	N	N	N	N	N	N	N	N	N	N	Y
Exposure was assessed measuring lead levels in blood or bone	Y	Y	Y	Y	Y	N	N	N	N	N	Y	Y	N	N	N	N	N	N	N	N	N	N	N	N	N	N	N	N	N	N
Outcomes were based on objective tests/standard criteria in ≥ 90% of study participants	Y	Y	N	N	N	N	Y	Y	N	N	Y	Y	N	N	N	N	N	N	N	N	N	N	N	N	N	N	N	N	N	N
Authors presented internal comparisons within study participants	Y	Y	Y	Y	Y	Y	Y	Y	Y	Y	Y	Y	Y	Y	Y	N	N	N	N	N	N	N	N	N	N	N	N	N	Y	Y
Authors controlled for relevant confounding factors in addition to age and sex^b^	Y	N	Y	Y	Y	N	N	N	Y	N	N	Y	N	N	N	N	N	N	N	N	N	N	N	N	N	N	N	N	N	N
Cohort studies																														
Loss to follow-up was independent of lead exposure	Y	Y	Y	Y	Y	—	—	—	—	—	—	—	Y	N	N	N	N	N	N	N	N	N	N	N	N	N	N	N	—	—
Intensity of search of disease was independent of lead exposure	Y	Y	Y	Y	Y	—	—	—	—	—	—	—	Y	N	N	N	N	N	N	N	N	N	N	N	N	N	N	N	—	—
Case–control and cross-sectional studies																														
Response rate among noncases was at least 70%	—	—	—	—	—	N	U	Y	U	N	U	Y	—	—	—	—	—	—	—	—	—	—	—	—	—	—	—	—	—	—
Exclusion criteria and data collection were similar for all participants	—	—	—	—	—	U	Y	Y	U	U	Y	Y	—	—	—	—	—	—	—	—	—	—	—	—	—	—	—	—	Y	Y
Non cases would have been cases if they had developed cardiovascular disease	—	—	—	—	—	U	N	Y	U	U	Y	Y	—	—	—	—	—	—	—	—	—	—	—	—	—	—	—	—	N	N
Interviewer was blinded with respect to the participant case or exposure status	—	—	—	—	—	U	U	U	U	U	U	Y	—	—	—	—	—	—	—	—	—	—	—	—	—	—	—	—	N	N

Abbreviations: —, not applicable; CC, case–control study; CS, cross-sectional study; N, no; PMS, proportional mortality study, Prosp., prospective; U, unclear; Y, yes.

a Criteria modified from Longnecker et al. (1988).

b In occupational studies, relevant factors included the healthy worker survivor effect. Studies that adjusted for blood pressure levels were considered not to fulfill this criterion.

## Appendix

**Appendix C:** Criteria for evaluating the design and data analysis of epidemiologic studies of lead exposure and intermediate cardiovascular end points.^a^

	Ventricular mass and function					Heart rate variability											Other cardiac abnormalities															Other vasc.
	Schwartz 1991	Zou et al. 1995	Tepper et al. 2001	Kasperczyk et al. 2005	Beck and Steinmetz-Beck 2005	Murata and Araki 1991	Teruya et al. 1991	Gennart et al. 1992	Murata et al. 1995	Ishida et al. 1996	Niu and Abbritti 1998	Böckelmann et al. 2002	Gajek et al. 2004	Andrzejak et al. 2004	Muzi et al. 2005	Jhun et al. 2005	Kosmider and Petelenz 1961	Kosmider 1962	Krotkiewski et al. 1964	Kosmider et al. 1965	Kosmider 1968	Stozinic and Colakovic 1980	Saric 1981	Kromhout et al. 1985	Kirkby and Gyntelberg 1985	Sroczynski et al. 1985	Shcherbak1988	Sroczynski et al. 1990	Gatagonova 1995 a,b,d	Gatagonova 1995 c	Cheng et al. 1998	Aiba et al. 1999
Association estimates based on lead assessed at the individual level	Y	Y	Y	N	N	N	Y	N	N	Y	N	N	N	N	N	Y	N	N	N	N	N	N	N	Y	N	N	N	N	N	N	Y	Y
Association estimates based on blood or bone lead measures	Y	Y	Y	N	N	N	Y	Y	N	Y	N	N	N	N	N	Y	N	N	N	N	N	N	N	Y	N	N	N	N	N	N	Y	Y
Cardiovascular tests were based on a standardized protocol	Y	Y	Y	Y	Y	Y	Y	Y	Y	Y	U	Y	Y	Y	Y	Y	N	N	Y	N	N	N	N	Y	Y	Y	U	Y	N	N	Y	N
Authors indicate that examiners received training to conduct cardiovascular tests	Y	N	Y	N	N	N	N	N	N	N	N	N	N	N	N	N	N	N	N	N	N	N	N	Y	Y	N	N	N	N	N	Y	U
Inclusion and exclusion criteria were similar for all participants	Y	U	Y	U	Y	Y	Y	N	Y	Y	U	Y	N	Y	U	Y	U	U	U	U	U	U	N	Y	Y	U	Y	U	U	U	Y	U
Recruitment procedures were similar for all participants	Y	U	Y	U	U	Y	Y	Y	N	Y	U	U	N	Y	U	Y	U	U	U	U	U	U	N	Y	Y	U	Y	U	U	U	Y	U
Response rate was at least 70%	Y	U	N	U	U	U	U	Y	U	U	U	U	U	U	U	U	U	U	U	U	U	U	U	Y	Y	U	U	U	U	U	Y	U
Examiner was blinded with respect to the participant exposure status	Y	U	U	U	U	U	Y	U	U	Y	U	U	U	U	U	Y	U	U	U	U	U	U	U	Y	N	U	U	U	U	U	Y	U
Authors controlled for relevant confounding factors in addition to age, sex	Y	Y	Y	N	N	N	N	N	N	N	N	N	N	N	N	N	N	N	N	N	N	N	N	Y	N	N	N	N	N	N	Y	N

Abbreviations: N, no; U, unclear; vasc., vascular; Y, yes;.

a Criteria modified from Appel et al. (2002).
